# Misinformation lingers in memory: Failure of three pro-vaccination strategies

**DOI:** 10.1371/journal.pone.0181640

**Published:** 2017-07-27

**Authors:** Sara Pluviano, Caroline Watt, Sergio Della Sala

**Affiliations:** 1 Department of Psychology, University of Edinburgh, Edinburgh, United Kingdom; 2 Suor Orsola Benincasa University, Naples, Italy; University College Cork, IRELAND

## Abstract

People’s inability to update their memories in light of corrective information may have important public health consequences, as in the case of vaccination choice. In the present study, we compare three potentially effective strategies in vaccine promotion: one contrasting myths vs. facts, one employing fact and icon boxes, and one showing images of non-vaccinated sick children. Beliefs in the autism/vaccines link and in vaccines side effects, along with intention to vaccinate a future child, were evaluated both immediately after the correction intervention and after a 7-day delay to reveal possible backfire effects. Results show that existing strategies to correct vaccine misinformation are ineffective and often backfire, resulting in the unintended opposite effect, reinforcing ill-founded beliefs about vaccination and reducing intentions to vaccinate. The implications for research on vaccines misinformation and recommendations for progress are discussed.

## Introduction

Vaccines are the safest and most effective tools for preventing infectious diseases and their success in achieving relevant public health outcomes, such as the reduction or eradication of many life-threatening conditions, is well-established. However, many people appear hesitant about vaccines, doubting their benefits, worrying over their safety or questioning the need for them. Addressing *vaccine hesitancy*, defined as a “delay in acceptance or refusal of vaccines despite availability of vaccination services” ([1], p575), is not a simple task for the following reasons. First, vaccine hesitancy is rooted in a set of cognitive mechanisms that conspire to render misinformation particularly “sticky” and pro-vaccination beliefs counter-intuitive [2], involving a multitude of emotional, social, cultural, and political factors [3]. Second, public information campaigns designed to dispel erroneous vaccination beliefs often overlook these factors and have limited or even unintended opposite effects [4, 5]. Furthermore, even when attempts to correct invalid information do not “backfire” by entrenching the original misinformation [6], they can frequently fail because people cannot successfully update their memories and still fall back on information they know is not correct in order to make inferences and explain events.

A vivid example of persistent reliance on mistaken beliefs despite extensive corrections involves the alleged risks of childhood vaccines, especially the purported link between certain vaccines and autism, fear of which escalated following the widely discredited Wakefield et al.’s study [7]. Despite an abundance of scientific evidence that shows no causal effect between any vaccine and autism [8, 9], sizable segments of the public still champion Wakefield’s view. To understand what causes such persistent reliance on patently incorrect information, it is useful to consider some relevant memory processes in more detail.

Classical laboratory research on memory for inferences [10, 6] demonstrates that the continued reliance on discredited information is very difficult to correct. Even when people clearly remember and understand a subsequent correction when asked about it immediately (suggesting that they have encoded it and can retrieve and potentially comply with it), they can still be influenced by the effect of the retracted misinformation. That is, people are susceptible to misinformation even though they had acknowledged that the information at hand is factually incorrect. As Rapp and Braasch stated ([11], p3), “the problem is not just that people rely on inaccurate information but that they rely on it *when they seemingly should know better*”. This seemingly irrational reliance on outright misinformation has been demonstrated with beliefs related to well-known material (e.g., biblical narratives [12, 13]), blatant hoaxes (e.g., paranormal claims [14]) or personally experienced events (e.g., distorted eyewitness testimonies [15]). It also occurs despite measures intended to make the presentation of information clearer and despite explicit warnings about the misleading nature of the information at hand [16, 17]. Therefore, simply retracting a piece of information does not stop its influence because outdated pieces of information linger in memory. In the case of vaccines, providing evidence about the safety of immunisation may not be enough as people may have heard or read somewhere that, for example, vaccines are not necessary, that they cause autism or contain dangerous chemicals. This false information persists in their minds.

An added complication is that the use of inaccurate information does not necessarily emerge immediately after its presentation but may occur after a delay between the initial presentation and later test points [18, 19]. Classical laboratory research on the lasting effect of misinformation in memory confirms that immediate tests following the presentation of inaccurate information are less likely to detect people’s susceptibility to misinformation compared to longer time intervals [20, 21].

Given these difficulties, there is the need to investigate debiasing practices used to disseminate correct information concerning vaccines, which, at the very least, should do no harm and ideally should help people better understand why and when they can trust scientific advice.

A number of strategies have been used to communicate the scientific consensus about vaccination and promote correction of misinformation. Perhaps the simplest strategy is exposing myths while concurrently debunking them, which is based on the idea of reiterating myths and then discrediting them with a number of facts. However, repeating myths might contribute to increasing their acceptance due to their perceived familiarity [22, 23, 24]. According to content-focused models of judgement, the strong arguments presented by the facts should decrease the acceptance of myths [25]. Moreover, some have argued that simply reviewing misinformation may even facilitate memory updating [26]. The scant available experimental evidence appears to only marginally support this approach. Consistent with it, in a natural classroom environment, Kowalski and Taylor [27] found that “refutational lectures” (in which popular misconceptions about psychology where directly addressed and contrasted with evidence opposing them and supporting the correct information) resulted in students having better access at the end of the course to the correct information. In the context of immunisation, there has been an inconclusive debate about whether health messages using a fact versus myth format are effective in reinforcing accurate information and refuting false information. Some studies found that repeating myths led to a marked increase in accepting those myths as true. For example, in an unpublished study, participants who read a “myths vs. facts” flyer regarding the flu vaccine subsequently misremembered myths as facts after only 30 minutes and expressed less favourable attitudes toward flu vaccination and lower intentions to get vaccinated, relative to control participants who did not read the flyer [25]. Similarly, in a study by Skurnik et al. [28] participants who read either true or false health-related statements could not distinguish fact from fiction after a 3-day delay. Even worse, the more they were warned that a statement was false, the more they accepted it as true. Despite these backfire effects, many pro-vaccination initiatives continue to confront erroneous beliefs with established evidence. A recent study by Cameron et al. [29] employing different facts versus myths message formats justifies the use of myths to overcome health misinformation, as participants who were exposed to facts, myths, and evidence to counteract those myths gained more knowledge regarding a specific health topic and had a better recall accuracy than those who were merely presented with factual information.

An alternative corrective technique is to represent information in visual form, using well-designed graphs which can attract and hold people’s attention, help the observer to process information more effectively, and facilitate recollection [30]. Being easier to process than complex verbal description, graphical representation of data can also provide more clarity and less opportunity for misinterpretation than text, simplifying complex ideas and highlighting what is important. According to Gigerenzer [31], a specific tool that could allow people with no medical or statistical competence to make competent decisions is a “fact box”, which consists of a table for transparent risk communication, summarizing the scientific evidence for a drug, treatment, or screening method in an easily understandable manner. Fact boxes usually show benefits and harms for people with and without treatment in plain frequencies, avoiding misleading statistics or statements of risk that may be misunderstood by laypeople. As transparent as a fact box, even though visually more appealing, is an “icon box”, which consists of a visual tool showing two groups of individuals: those who underwent a treatment and those who did not. Each individual is represented by an icon indicating benefits and harms. Although there has been relatively little research on the use of fact boxes as tools for informing the general public about pros and cons of a treatment, some studies show promising results. For instance, Schwartz et al. [32, 33] demonstrated how the use of simple fact boxes about drugs may help people to make better informed choices by improving their knowledge of drug benefits and side effects.

A third alternative to counter anti-vaccination attitudes is to harness the power that fear exerts on people, by highlighting grave risks from diseases (i.e., emotionally-charged messages that show the possible serious consequences of a disease likely to happen in non-vaccinated individuals). According to Nyhan et al. [4], this puts individuals into the “domain of losses”, making them aware of the dangers associated with the decision not to vaccinate their own children. An early meta-analysis by Witte and Allen [34] revealed that fear appeals can actually increase the perception of the severity of the health threat inducing behaviour changes, at least as long as the individuals believe that they are able to protect themselves and avert the threat. Results from a comprehensive meta-analysis by Tannenbaum et al. [35] showed that fear appeals are particularly effective at positively influencing attitudes, intentions, and behaviour under specific circumstances. Powerful persuasive messages like fear appeals seem also more successful when they recommend one-time behaviours (as is often the case for vaccination) compared with behaviours that should be repeated over an extended period of time [36, 37]. However, the outcome of Nyhan et al.’s [4] recent study did not corroborate the effectiveness of this approach. The images of sick children that they used to make risks more salient paradoxically increased beliefs in the false vaccine/autism link, a phenomenon they labelled danger-priming effect. Similarly, a study by Guillaumier et al. [38] on the effectiveness of diverse anti-smoking messages showed that highly emotive warnings, which stressed the negative health effects of smoking via resonant texts and vivid pictures, did not motivate smokers to quit their behaviour. To achieve this aim, the use of a plain cigarette packaging proved to be more effective [39].

Notwithstanding the centrality of the vaccine issue, there is little systematic research on correcting vaccine misinformation and we found no strong evidence recommending any specific intervention to address vaccines hesitancy [3, 40, 41]. Therefore, the present study aims at adding to our knowledge by directly contrasting the effectiveness of three promising strategies employed to reduce vaccine misperceptions in a controlled experimental setting. More specifically, we sought to test the three information strategies discussed above, namely the myth vs. fact message frame, the presentation of fact/icon boxes, and the use of fear appeals. Two research questions were posed: 1) to what extent can these different approaches influence people’s vaccination beliefs and behaviour?; 2) does any of these approaches have a comparative advantage in terms of its ability to counter anti-vaccination attitudes, given the persistent effect of misinformation in influencing memory, reasoning and decision making, as well as the possibility of backfire effects? Finding answers to these questions can help to determine the best way to provide corrective information, undermining widespread vaccination myths, advancing our understanding of how people process information regarding controversial health issues, and ultimately improving public health.

## Method

### Participants

Participants were students from diverse departments of the University of Edinburgh, the Suor Orsola Benincasa University of Naples, and the Second University of Naples, resulting in an initial sample of 134 individuals. Participants were recruited via adverts, e-mail invitations, and snowball sampling. All participants gave their written informed consent and participated on a voluntary basis. The first wave of the study was completed by 134 respondents. We then re-contacted all participants from Wave 1. A total of 120 participants completed the second wave of the study (drop-out rate: 10.%). Respondents who dropped out from the study and those who completed both waves did not differ on any relevant aspect (a complete report can be provided by the authors upon request). Our sample for all subsequent analyses consisted of the 120 participants who completed both waves of the study. Among those, 47 (39.2%) were men and 73 (60.8%) women. Mean age was 25.35 years (*SD* 3.52, range 19–34). Most participants had a Bachelor’s (n. 46, 38.3%) or a Masters’ degree (n. 63, 52.5%), while 11 respondents (9.2%) were PhD students. The study received ethical approval from the Psychology Research Ethics Committee of the University of Edinburgh. Data collection was conducted from March to June, 2016.

### Materials

#### Questionnaires

All participants in the study completed two questionnaires. The first questionnaire was a preliminary survey aimed at assessing participants’ baseline beliefs and attitudes towards vaccines, which has been used in previous studies [42, 4]. It consisted of 8 items which covered common attitudes from both the pro- (e.g., “Getting vaccines is a good way to protect my future child(ren) from disease”) and the anti-vaccination side (e.g., “Some vaccines cause autism in healthy children”). Participants were asked to indicate their degree of agreement with each statement on a 5-point Likert scale, ranging from 1 (= strongly disagree) to 5 (= strongly agree). The second questionnaire was a post-manipulation survey that assessed whether and how participants’ beliefs and attitudes toward vaccines changed compared to the baseline measure. The three-item post-manipulation survey has also been used in previous studies [4]. The first item evaluated general misperceptions about vaccines causing autism (“Some vaccines cause autism in healthy children”) with a 5-point Likert scale, ranging from 1 (= strongly disagree) to 5 (= strongly agree). Item two investigated beliefs about vaccines side effects (“Children vaccinated against measles, mumps, and rubella will suffer serious side effects”) with a 6-point Likert scale, ranging from 1 (= very unlikely) to 6 (= very likely). The third item asked participants to evaluate how likely they would be to give MMR vaccine to their child on a 6-point Likert scale, ranging from 1 (= very unlikely) to 6 (= very likely). This post-manipulation survey was administered twice, immediately after the correction interventions ('Time 1') and after a 7-day delay to evaluate the longevity and robustness of the observed effects ('Time 2').

#### Correction interventions

**Fig 1:**
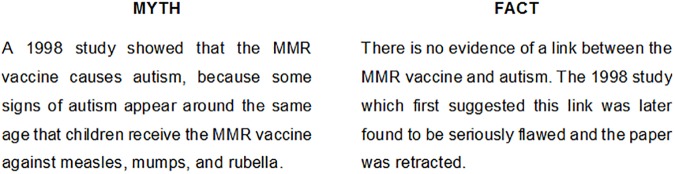
Example of message for the Myths vs. Facts Correction.

**Fig 2:**
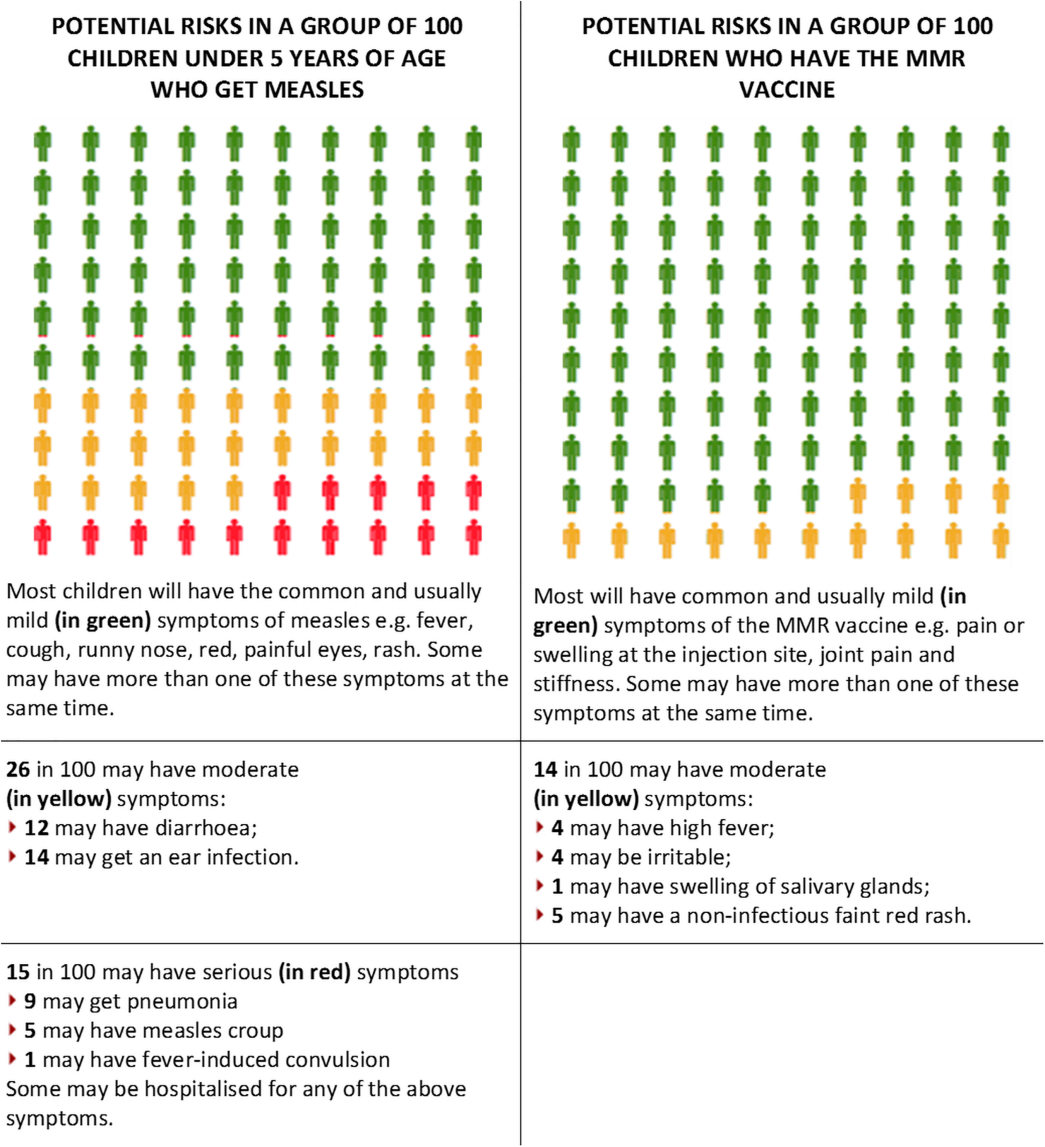
Example of message for the Visual Correction. This message compared the potential problems caused by measles with the potential problems caused by the MMR vaccine. Common and usually mild symptoms that can be treated at home are represented in green, moderate complications that need medical attention but may not include hospitalisation are portrayed in yellow, and serious complications that need urgent medical attention and could include hospitalisation are marked in red.

**Fig 3:**
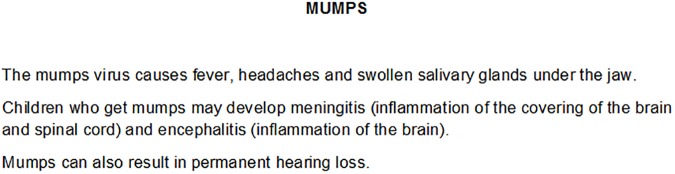
Example of message for the Fear Correction. This message was accompanied by a picture of a child with swelling at the side of the face under the ears.

Participants could be exposed to one of 4 experimental conditions. Examples of messages for each condition are presented in Figs 1, 2 and 3. In the first condition (*Myths vs*. *Facts Correction*), participants received a booklet confronting 10 “myths” with a number of “facts”. Each page of the leaflet contrasted a popular erroneous belief about vaccination (e.g., low perception of vaccine efficacy, safety concerns about immune overload, fear of the alleged presence of toxic poisons and chemicals in vaccines, etc.) with established evidence intended at decreasing the acceptance of that myth. Myth/fact #10 specifically addressed the common misconception about the link between MMR vaccine and autism (Fig 1). The text for this intervention, which was taken nearly verbatim from the WHO’s (World Health Organization) website, was displayed in a columnar format, with the “myth” and “fact” headings on each column to avoid any ambiguity. The length of each myth and fact was matched to reduce the risk of individuals devoting more attention to one text rather than the other.

In the second condition (*Visual Correction*), participants viewed a series of tables comparing the potential problems caused by measles, mumps, and rubella with the potential side effects caused by the MMR vaccine (Fig 2). Each table showed the chance of various outcomes for people who do and do not get vaccinated. In order to test the effectiveness of graphic material as a communication device, the MMR Decision Aid presented in the NCIRS’ (National Centre for Immunization Research & Surveillance) website was adapted for this intervention.

In the third condition (*Fear Correction*) participants were presented with pictures of unvaccinated children with measles, mumps, and rubella, along with the description of the symptoms of each disease and a brief warning about the importance of vaccinating one’s own child (Fig 3). Materials for this intervention were drawn from the IDPH’s (Illinois Department of Public Health) website. The brief warning in the instructions, formulated in personalised language (i.e., “The following images show some of the consequences *you* may face choosing to not vaccinate *your* child”), emphasized the similarities between the victims of these diseases and participants’ actual or future children to increase perceptions of susceptibility.

Finally, in the control condition (*Control*) participants read two unrelated fact sheets containing tips to help prevent medical errors [43] and get safer healthcare [44]. All the intervention materials are available in S1 File.

### Procedure

**Fig 4:**
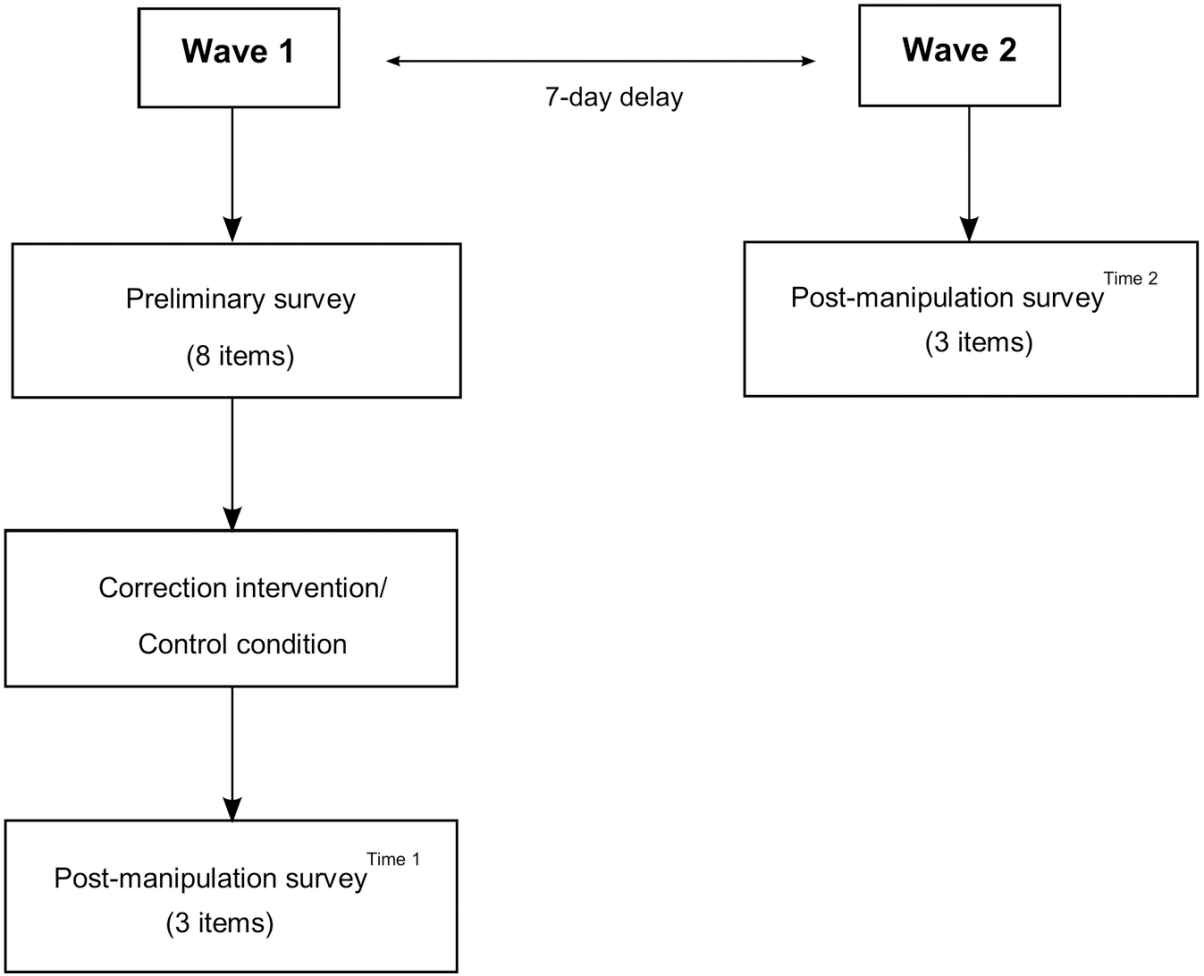
Overview of the different phases of the study.

Participants were informed at the outset that the experiment consisted of two parts, both seeking to gather their opinions about vaccines (Fig 4). After providing some demographic details (i.e., sex, age, educational level), they completed the preliminary survey. Next, they were randomly assigned to 1 of the 4 conditions. Participants were unaware of the other experimental conditions and researchers were blind to condition allocation until printed materials were delivered to the study participants. After the experimental intervention, participants completed the post-manipulation survey (*Time 1*), evaluating their beliefs in the link between vaccines and autism, in vaccines side effects, and vaccination intention. After a 7-day delay, they participated in the second wave of the study during which the same questions of the post-manipulation survey were asked again (*Time 2*). Then, participants were carefully debriefed. The researcher made the actual purpose of the study clear, revealing the experimental condition to which each participant was assigned and asking whether he/she had any questions about the study. Lastly, participants were thanked and compensated.

Three key outcomes were evaluated: individual beliefs in vaccines causing autism (*Vaccines Cause Autism*) and side effects (*Vaccines Side Effects*), and intention to vaccinate (*Vaccine Hesitancy*). As these outcomes were collected twice for all the participants, our study design consisted of one between-subject variable (*Correction Intervention*) with four levels (*Myths vs*. *Facts Correction*, *Visual Correction*, *Fear Correction*, and *Control*), and three within-subject variables (*Vaccines Cause Autism*, *Vaccines Side Effects*, and *Vaccine Hesitancy*), each with two levels (*Time 1* and *Time 2*).

## Results

Data were stored and analysed using SPSS (version 20). The dataset is available in S2 File.

For ease of interpretation, the item evaluating one’s vaccination intent was reverse-coded so that higher values indicated higher vaccine hesitancy. Therefore, all 3 key outcomes were in the same direction as higher means indicated stronger vaccine misconceptions.

To determine whether correction interventions had a time-varying effect or resulted in null effect on vaccination attitudes, different mixed-design ANOVAs were performed on the whole sample, with independent measure on correction interventions (treated as a between-subjects variable) and repeated dependent measures on the three items/outcomes of the post-manipulation survey, i.e., *Vaccines Cause Autism*, *Vaccines Side Effects*, and *Vaccine Hesitancy* (treated as within-subjects variables). When significant interactions between correction interventions and time were found, separate estimates of simple main effects were carried out. Finally, to test whether the difference between outcomes measurements at Time 1 and Time 2 was statistically significant, we created 'change scores' for each of the 3 key outcomes (*Vaccines Cause Autism*, *Vaccines Side Effects*, and *Vaccine Hesitancy*), which were computed as the difference between mean outcomes scores at Time 2 and Time 1.

For multiple comparisons between groups, Tukey’s HSD correction method was applied. Significance was accepted at *p* < .05 for all statistical analyses.

Table 1 indicates means and standard deviations for the outcomes at Time 1 and Time 2 in the whole sample. Following Table 1, the three sub-sections address our three key outcome measures.

**Table 1:** Descriptive statistics (means and standard deviations) for the outcomes at Time 1 and Time 2.

	Time 1		Time 2	
	*M*	*SD*	*M*	*SD*
Vaccines Cause Autism	1.8	.9	2.12	.93
Vaccines Side Effects	2.1	1	2.72	1.3
Vaccine Hesitancy	1.66	.83	2.02	.92

### Beliefs in vaccines/autism link

**Fig 5:**
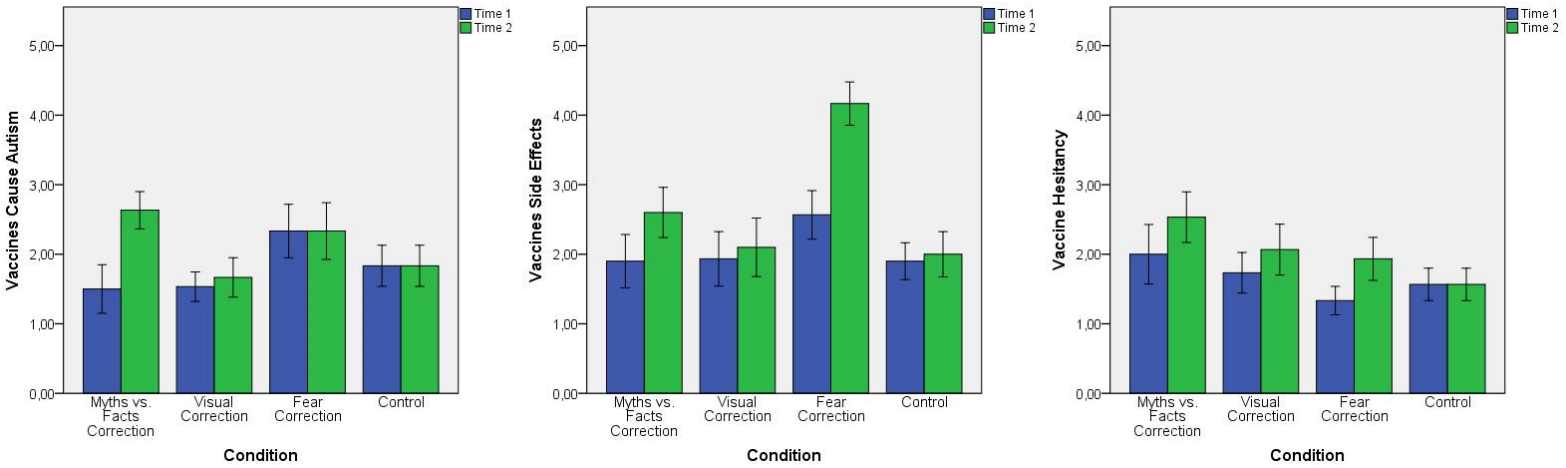
**Mean scores of the 3 key outcomes evaluated: *Vaccines Cause Autism* (A), *Vaccines Side Effects* (B), and *Vaccine Hesitancy* (C) by condition and time (after a week).** Outcomes means at Time 1 are represented by blue bars, while outcome means at Time 2 by green bars. Error Bars: 95% CI.

Concerning beliefs in the vaccines/autism link, there was a statistically significant interaction between correction interventions and time [F(3, 116) = 23.263, *p* < .001, partial η^2^ = .376]. The data in the conditions by time interaction are detailed in Fig 5A. Simple main effect for condition revealed that there was a statistically significant difference in these beliefs between interventions at Time 1 [F(3, 116) = 6.183, *p* = .001, partial η^2^ = .138]. Indeed, beliefs in the vaccines/autism link were significantly greater in the fear correction intervention compared to the myths vs. facts (M = .83, SE = .22, *p* = .001) and visual condition (M = .8, SE = .22, *p* = .002). A statistically significant difference in these beliefs between interventions was also detected at Time 2 [F(3, 116) = 8.194, *p* < .001, partial η^2^ = .175]. This time, beliefs in the vaccines/autism link were statistically significantly higher in the myths vs. facts condition compared to the visual (M = .97, SE = .22, *p* < .001) and control condition (M = .8, SE = .22, *p* = .002), and in the fear condition compared to visual condition (M = .67, SE = .22, *p* = .016). Simple main effect for time confirmed that there was a statistically significant effect of time on beliefs in the vaccines/autism link for the myths vs. facts [F(1, 29) = 31.508, *p* < .001, partial η^2^ = .521] and the visual condition [F(1, 29) = 4.462, p = .043, partial η^2^ = .133]. Pairwise comparisons indicated that these beliefs were statistically significantly higher at Time 2 compared to Time 1 for both the myths vs. facts (M = 1.13, SE = .2, *p* < .001) and visual condition (M = .13, SE = .06, *p* = .043).

**Fig 6:**
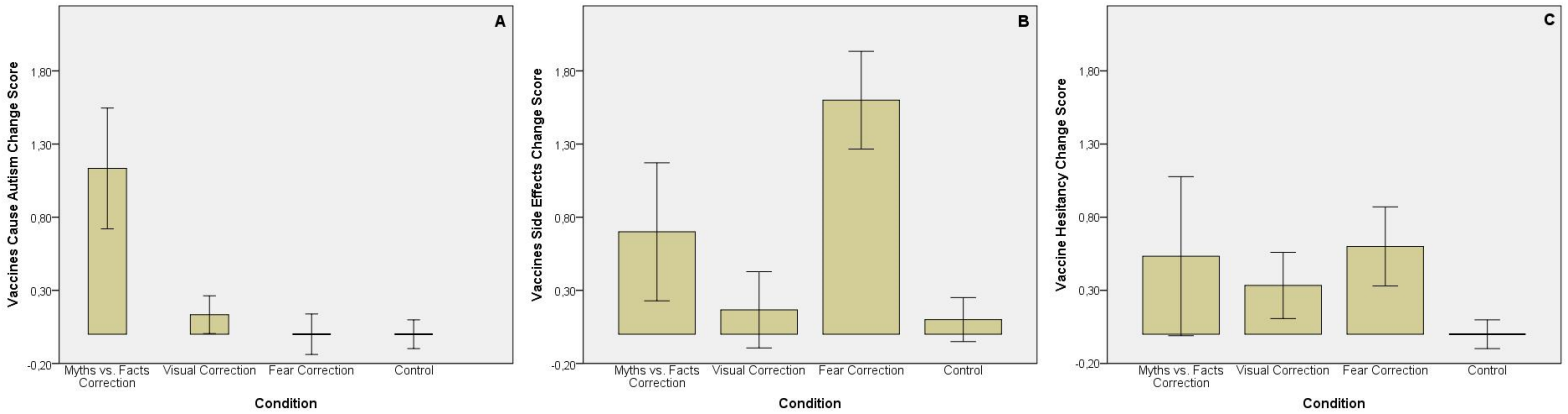
**Change scores for the 3 key outcomes evaluated: *Vaccine Cause Autism Change Score* (A), *Vaccines Side Effects Change Score* (B), and *Vaccine Hesitancy Change Score* (C) across conditions.** Error Bars: 95% CI.

As shown in Fig 6A, there was a significant difference between conditions in *Vaccine Cause Autism Change Score* [F(3,116) = 23.263, *p* < .001]. This effect was driven by the myths vs. facts condition, which led to larger changes in scores and therefore strongest beliefs in vaccines causing autism compared to the other two correction interventions, that is the visual (M = 1, SE = .16, *p* < .001) and the fear condition (M = 1.13, SE = .16, *p* < .001), and the control condition (M = 1.13, SE = .16, *p* < .001).

### Beliefs in vaccines side effects

Concerning beliefs in vaccines side effects, there was a statistically significant interaction between interventions and time [F(3, 116) = 18.914, *p* < .001, partial η^2^ = .328]. The data in the conditions by time interaction are detailed in Fig 5B. Simple main effect for condition revealed that there was a statistically significant difference in these beliefs between interventions at Time 1 [F(3, 116) = 3.651, *p* = .015, partial η^2^ = .086]. Beliefs in vaccines side effects were statistically significantly higher in the fear correction intervention compared to the myth vs. facts (M = .67, SE = .24, *p* = .035), visual (M = .63, SE = .24, *p* = .05), and control condition (M = .67, SE = .24, *p* = .035). A statistically significant difference in beliefs concerning vaccines side effects between interventions was also detected at Time 2 [F(3, 116) = 32.919, *p* < .001, partial η^2^ = .46]. Again, beliefs in vaccines side effects were statistically significantly greater in the fear correction intervention compared to the myth vs. facts (M = 1.57, SE = .25, *p* < .001), visual (M = 2.07, SE = .25, *p* < .001), and control condition (M = 2.17, SE = .25, *p* < .001). Simple main effect for time revealed that there was a statistically significant effect of time on beliefs in vaccines side effects for the myths vs. facts [F(1, 29) = 9.207, *p* = .005, partial η^2^ = .241] and fear intervention [F(1, 29) = 96.000, *p* < .001, partial η^2^ = .768]. Pairwise comparisons indicated that these beliefs were statistically significantly higher at Time 2 compared to Time 1 for both the myths vs. facts (M = .7, SE = .23, *p* = .005) and fear condition (M = 1.6, SE = .16, *p* < .001).

There was also a significant difference between conditions in *Vaccines Side Effects Change Score* [F(3,116) = 18.914, *p* < .001] (Fig 6B). This time, this effect was driven by the fear condition, which led to larger changes in scores and therefore strongest beliefs in vaccines causing side effects than the other two correction interventions, that is the myths vs. facts (M = .9, SE = .22, *p* = .001) and the visual condition (M = 1.43, SE = .22, *p* < .001), and the control condition (M = 1.5, SE = .22, *p* < .001).

### Vaccine hesitancy

Concerning vaccine hesitancy, there was a statistically significant interaction between interventions and time [F(3, 116) = 2.828, *p* = .042, partial η^2^ = .068]. The data in the conditions by time interaction are detailed in Fig 5C. Simple main effects for condition revealed that there was a statistically significant difference in vaccination intentions between interventions at Time 1[F(3, 116) = 3.613, *p* = .015, partial η^2^ = .085]. Vaccine hesitancy was statistically significantly higher in the myths vs. facts condition compared to the fear condition (M = .67, SE = .21, *p* = .01). A statistically significant difference in vaccination hesitancy between interventions was also detected at Time 2 [F(3, 116) = 6.413, *p* < .001, partial η^2^ = .142]. Vaccine hesitancy was statistically significantly higher in the myths vs. facts condition compared to the fear (M = .6, SE = .22, *p* = .04) and control condition (M = .97, SE = .22, *p* < .001). Simple main effects for time showed that there was a statistically significant effect of time on vaccination intentions for the fear condition [F(1, 29) = 20.605, *p* < .001, partial η^2^ = .415]. Pairwise comparisons indicated that vaccine hesitancy increased over time (M = .6, SD = .13, *p* < .001).

There was a significant difference between conditions in *Vaccine Hesitancy Change Score* [F(3,116) = 2.828, *p* = .042], (Fig 6C) as the fear correction led to larger changes in scores and higher vaccine hesitancy than the control condition (M = .6, SE = .23, *p* = .045).

## Discussion

The serious psychological and social implications of the persistence of incorrect information have been under investigation for decades, although interest has intensified in recent years, arguably because of the increasing presence of misinformation regarding relevant and sensitive topics, such as health care. The present study addressed the pertinent case of misinformation about vaccines. The correction of vaccine misinformation has become an urgent priority to assure the continued success of immunization programs. In this respect, some authors advocated the need to carefully test pro-vaccination messaging before making it public, especially given the risk of backfire effects, whereby messages created with a pro-social intent can result in the targeted attitude or behaviour at issue actually becoming worse [4, 5]. However, there has been little systematic comparison of different forms of correction of vaccine misinformation.

In this study, we provided a direct test of corrections on factual beliefs about vaccines, investigating the different impact of three common strategies used to promote vaccination. These were the use of the myth vs. fact message frame, the presentation of fact/icon boxes, and a format involving fear-inducing material. We outlined two core questions to evaluate the continued influence effect of misinformation. The first question concerned the extent to which these different techniques supported people’s updating process of information, being effective in discounting or at least reducing vaccine misperceptions. The second question was constrained by the answer to the previous one and reconnected to the issue of memory: in other words, to what extent the various effects described in this study faded, were amplified, or backfired over time? As our primary concern was in the phenomenon by which misinformation in memory can affect later inferences and behaviours, we incorporated a delay in our design to evaluate the effectiveness of the aforementioned strategic messages over time, with a particular interest in explaining possible backfire effects.

Our study provided further support to the growing literature showing how corrective information may have unexpected and even counter-productive results. Specifically, we found that the myths vs. facts format, at odds with its aims, induced stronger beliefs in the vaccine/autism link and in vaccines side effects over time, lending credit to the literature showing that countering false information in ways that repeat it may further contribute to its dissemination [25]. Also the exposure to fear appeals through images of sick children led to more increased misperceptions about vaccines causing autism. Moreover, this corrective strategy induced the strongest beliefs in vaccines side effects, highlighting the negative consequences of using loss-framed messages and fear appeals to promote preventive health behaviours [45, 38]. Our findings also suggest that no corrective strategy was useful in enhancing vaccination intention. Compared to the other techniques, the usage of fact/icon boxes resulted in less damage but did not bring any effective result.

Our pattern of results thus confirms that there should be more testing of public health campaign messages. This is especially true because corrective strategies may appear effective immediately yet backfire even after a short delay, when the message they tried to convey gradually fades from memory, allowing common misconceptions to be more easily remembered and identified as true [25]. This is the case of one of the most frequently used corrective strategy employing the myths versus facts format, which often backfires because the simple repetition of the myth, though well-intended and necessary in order to contrast it with the available evidence, paradoxically amplifies the familiarity of that false claim making it seem even more believable and widely-shared [46]. This happens, at least partly, because people tend to mistake repetition for truth, a phenomenon known as the “illusory truth” effect [22, 23]. Familiarity appears as a key determinant of this effect; indeed, when something seems familiar is easier to process and one is more inclined to believe it [24], regardless of whether the statement is factually true or false [22, 47] or was initially rated as credible or questionable [48].

Multiple explanations have been proposed for the continued influence of misinformation. A strong argument is that, once a belief is formed, people generate explanations that fit and further reinforce this belief and tend to vigorously reject counter-arguments that make them uncomfortable, regardless of their validity [49]. People’s worldview, or personal ideology, can indeed override unwelcome facts and determine the effectiveness of retractions, which can even backfire when they are attitude-incongruent and strengthen the initial held beliefs (i.e., “attitude bolstering” [50]). This effect can be better understood within a cognitive consistency perspective [51], according to which rejecting a belief would generate numerous inconsistencies that threaten one’s self-concept. To reduce the emerging fear they may feel, people (especially those with high personal or partisan stakes on the issue in question) can engage in different defensive mechanisms, which are likely to appear in combination and may include selective exposure (engaging in a biased search process, seeking out information from outlets that supports ones’ preconceptions), source derogation (dismissing the validity of the source of the unwelcome corrective information), social validation (bringing to mind others who held the same view), and even reactance (coming to support one’s original opinion even more strongly, which is a classic backfire effect).

Another explanation for the lingering effect of misinformation assumes that people build mental models of unfolding events. If a central piece of the model is invalidated, people are left with a gap in their knowledge of the event, whose representation simply does not make any sense unless one decides to maintain the false and invalidated information. Thus, feeling uncomfortable with gaps in their understanding, people prefer a more readily available and complete model, albeit inaccurate, over a correct but incomplete one, sticking to the original idea and ignoring the retraction [52].

Presumably, a golden strategy capable of overcoming all the intricacies of setting people straight, regardless of their basic beliefs and/or temporal shifts, does not exist. Public information campaigns may instead benefit from tailoring different, simultaneous, and frequent interventions to increase the likelihood of corrective messages’ dissemination and acceptance [40]. Ideally, corrective strategies should be directed at the precise factors that may influence vaccination decision-making and impede vaccine uptake, which include, over and beyond strong attitudes against vaccines, social norms pushing individuals to conform to the majority’s behaviour, standards for vaccine uptake in a specific population, and structural barriers to vaccination such as potential financial costs of vaccines and their ease of access. Successful interventions should therefore be targeted to differently “driven” vaccine-hesitant individuals. For instance, when people do not vaccinate because they lack confidence in vaccines, corrective strategies should dispel vaccination myths, or when people do not vaccinate because perceived risks outweigh benefits, interventions should emphasize the social benefit deriving from vaccination and add incentives [41]. However, the inter-relationship of multi-level factors which contribute to vaccine hesitancy seems somewhat difficult to disentangle in order to make such targeted approach successful; indeed, the independent and relative impact of each determinant of vaccination choice is complex and context-specific, varying across time, place, and vaccines [3]. What is clear, though, is the urgent need for appropriately designed, well-executed, and rigorously evaluated interventions to address parental vaccine refusal and hesitancy [53].

Some aspects of our experimental procedures may limit the generalization of the findings. Firstly, we used a convenience sample with limited variability in age and educational level. Future research should rely on more representative and specific samples (e.g., parents, health practitioners), as well as investigate possible moderating variables (e.g., age, education, socioeconomic status) to account for a broader range of individual differences in how people comprehend scientific information. Also, our participants were domiciled in Italy or Scotland, which raises the question of possible effects of between-country heterogeneity in vaccine attitudes, which might introduce uncontrolled variance into our data. To the best of our knowledge, comparative data on vaccine attitudes and uptake in the UK and Italy are still lacking. However, some studies report a general shift towards a more positive perception of vaccines in both countries [54, 55]. Because our participants were randomly allocated to conditions, there is no reason to expect our results to be systematically biased by between-country heterogeneity. Because of the small sample size of our study, we are not able to ascertain whether these differences actually exist and are reflected in our findings. Secondly, self-reported vaccine uptake should be supplemented with objective data from primary records to produce a more reliable measure of uptake. Moreover, as beliefs can change and evolve dynamically over time, prospective longitudinal data are also needed to assess the robustness of changes in individual beliefs.

Notwithstanding these limitations, our findings offer a useful example of how factual information is misremembered over time. More importantly, our work can help public health authorities and practitioners to understand why it is necessary to adopt an appropriate strategy to influence people’s beliefs and behaviours toward vaccination, which can result in better health outcomes for the individuals themselves and for society as a whole.

## Supporting information

S1 File Intervention materials. This file includes all the intervention materials employed in this study: the preliminary and post-manipulation survey, the Myths Vs. Facts Correction, the Visual Correction, the Fear Correction, and the Control Condition.
(PDF)

S2 File Dataset. (XLSX)
