# Supplementary material for: Misinformation lingers in memory: Failure of three pro-vaccination strategies
Source: PLoS One. 2017 Jul 27;12(7):e0181640. doi: 10.1371/journal.pone.0181640 (PMC5547702; doi:10.1371/journal.pone.0181640)
Supplement: S1 File — This file includes all the intervention materials employed in this study: the preliminary and post-manipulation survey, the Myths Vs. Facts Correction, the Visual Correction, the Fear Correction, and the Control Condition. (PDF) [file pone.0181640.s001.pdf]

PRELIMINARY SURVEY

Sex:	
Age:	
Educational level:	

First, please put your sex, age, and educational level on the top of this page. Then, please read the following statements and check the box (<u>one</u> for each row) which best indicates your idea and/or behaviour.

	**1 = Strongly Disagree**	**2 = Disagree**	**3 = Neutral**	**4 = Agree**	**5 = Strongly Agree**
Getting vaccines is a good way to protect my future child(ren) from disease.	☐	☐	☐	☐	☐
Generally I would do what my doctor recommends about vaccines for my future child(ren).	☐	☐	☐	☐	☐
New vaccines are recommended only if they are as safe as older vaccines.	☐	☐	☐	☐	☐
My future child(ren) will not need vaccines for diseases that are not common anymore.	☐	☐	☐	☐	☐
Parents should have the right to refuse vaccines that are required for school for any reason.	☐	☐	☐	☐	☐
So many children are vaccinated that my future child(ren) will be safe from these illnesses even if I will not vaccinate them.	☐	☐	☐	☐	☐
I am concerned about serious adverse effects of vaccines.	☐	☐	☐	☐	☐
Some vaccines cause autism in healthy children.	☐	☐	☐	☐	☐

POST-MANIPULATION SURVEY

Now, please answer the following questions. For each item, check the box which best indicates your idea and/or behaviour.

	1 = Strongly Disagree	2 = Disagree	3 = Neutral	4 = Agree	5 = Strongly Agree
Some vaccines cause autism in healthy children.	☐	☐	☐	☐	☐

	1 = Very Unlikely	2 = Somewhat Unlikely	3 = Slightly Unlikely	4 = Slightly Likely	5 = Somewhat Likely	6 = Very Likely
Just based on what you know, how likely is it that children who get the measles, mumps, and rubella vaccine – which is known as the MMR vaccine – will suffer serious side effects?	☐	☐	☐	☐	☐	☐
How likely is that you would give your future child(ren) the measles, mumps, and rubella vaccine, which is known as the MMR vaccine?	☐	☐	☐	☐	☐	☐

Finally, please indicate your email address and a password that we will use to match your answers in the first and second session of the study. Be sure to remember it for the second time we will meet again.

Email address:
Password:

Myths Vs. Facts Correction

IN THIS EXPERIMENT YOU WILL READ A SERIES OF STATEMENTS THAT CONTRAST MYTHS AND FACTS ABOUT VACCINES. YOU CAN GO THROUGH THEM AT YOUR OWN PACE. BEGIN ON THE NEXT PAGE.

1

MYTH vs. FACT

MYTH

It is well-known that better hygiene, sanitation, and nutrition are actually responsible for decreased infections, not vaccines. Therefore, vaccines are not necessary.

FACT

Many infections can spread regardless of improved sanitation. If people are not vaccinated, diseases that have become uncommon, such as polio, will quickly reappear.

MYTH vs. FACT

Vaccines are risky. They have several damaging and long-term side-effects that are as yet unknown. Vaccination can even be fatal.

Vaccines are very safe. You are far more likely to be seriously injured by a vaccine-preventable disease than by a vaccine.

3

MYTH vs. FACT

The combined vaccine against diphtheria, tetanus and pertussis (*whooping cough*) and the vaccine against poliomyelitis cause *sudden infant death syndrome* (SIDS).

There is no link between giving these vaccines and *sudden infant death*. It is only coincidental that these vaccines are administered at a time when babies can suffer SIDS.

4

MYTH vs. FACT

MYTH: Many diseases that can be preventable by vaccines, such as meningitis, are almost eradicated in my country. Therefore, there is no reason to be vaccinated, especially for uncommon diseases.

FACT: In a highly inter-connected world, the infectious agents that cause diseases, even if uncommon, continue to circulate, can easily cross geographical borders, and infect anyone who is not protected.

5

MYTH

vs.

FACT

Vaccine-preventable childhood illnesses are not so serious. They are just an unfortunate fact of life that everyone has to face.

Illnesses such as measles, mumps and rubella are serious and can lead to severe complications, such as encephalitis.

6

MYTH vs. FACT

MYTH

Immunisation schedules are extremely daunting. Giving a child more than one vaccine at a time can overwhelm his or her immune system and increase the risk of harmful side-effects.

FACT

Giving several vaccines at the same time has no adverse effect. Rather, it implies fewer injections. Also, children are more likely to complete the recommended vaccinations on schedule.

7

MYTH	vs.	FACT
Influenza, like several other common diseases, is not a big deal for most people. It is just a nuisance and the vaccine isn't very effective.		Influenza is a serious disease that can even be fatal. Pregnant women, children, elderly are at higher risk of severe infection and death.

8

MYTH vs. FACT

Natural immunity is better than vaccine-acquired immunity. Indeed, catching a disease and then getting sick results in a stronger immunity to the disease than a vaccination.

Vaccines interact with the immune system to produce a response similar to that produced by the natural infection, but they protect against its potential severe complications.

9

MYTH vs. FACT

Aside from antigens and antibiotic, we do not know what goes into a vaccine. They can contain dangerous toxic chemicals, such as *thiomersal*.

Vaccines are safe. For example, there is no evidence to suggest that the amount of *thiomersal* contained in vaccines poses a health risk.

MYTH vs. FACT

A 1998 study showed that the MMR vaccine causes autism, because some signs of autism appear around the same age that children receive the *MMR vaccine* against measles, mumps, and rubella.

There is no evidence of a link between the MMR vaccine and autism. The 1998 study which first suggested this link was later found to be seriously flawed and the paper was retracted.

Visual Correction

- IN THIS EXPERIMENT YOU WILL EXAMINE A SERIES OF TABLES WHICH COMPARE THE NEGATIVE CONSEQUENCES OF SOME DISEASES SUCH AS MEASLES, MUMPS AND RUBELLA WITH THE POTENTIAL PROBLEMS CAUSED BY THE *MMR* VACCINE, INJECTED TO PREVENT THESE DISEASES.

- YOU CAN GO THROUGH THEM AT YOUR OWN PACE.

- BEGIN ON THE NEXT PAGE.

- ***What are measles, mumps and rubella?***

Measles, mumps and rubella are infectious diseases that are caused by three different viruses. They are spread when the viruses are passed from an infectious person to someone who is not immune to them. Rubella is also known as "German measles".

- ***What is the MMR vaccine?***

MMR is the combined vaccine against measles, mumps and rubella. It contains live, weakened measles, mumps and rubella viruses. Over 90 countries around the world use MMR vaccine. Two doses of the vaccine are usually recommended to be given early in life.

Measles

- ***Common symptoms of measles***

These are usually mild symptoms and include fever, loss of appetite, rash, diarrhoea, runny nose, cough and red painful eyes. Children who get measles usually have to spend about 5 days in bed and have to take 10 to 14 days off from school, if there is no serious complication.

- ***Complications of measles***

These are usually serious conditions and include ear infections, pneumonia, fits or convulsions, croup, inflammation of the brain (encephalitis), which could result in hospitalisation. A late complication of measles is the so-called subacute sclerosing panencephalitis (SSPE), which causes progressive brain damage and nearly always results in death.

Now look at the following table that compares the potential problems caused by measles with the potential problems caused by the MMR vaccine.

Green - Common, usually mild symptoms that can be treated at home.
Yellow - Moderate complications that need medical attention but may not include hospitalisation.
Red - Serious complications that need urgent medical attention and could include hospitalisation.

**POTENTIAL RISKS IN A GROUP OF 100 CHILDREN UNDER 5 YEARS OF AGE WHO GET MEASLES**	**POTENTIAL RISKS IN A GROUP OF 100 CHILDREN WHO HAVE THE MMR VACCINE**
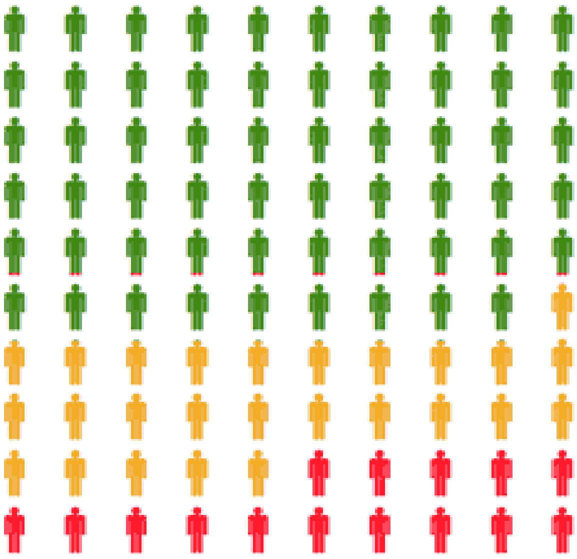 Most children will have the common and usually mild **(in green)** symptoms of measles e.g. fever, cough, runny nose, red, painful eyes, rash. Some may have more than one of these symptoms at the same time.	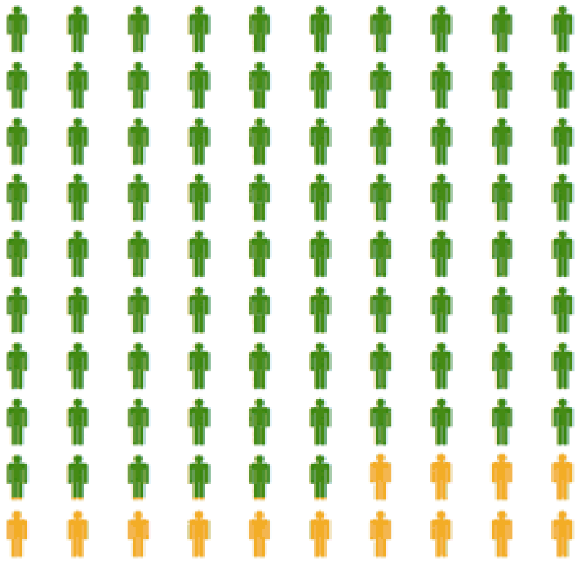 Most will have common and usually mild **(in green)** symptoms of the MMR vaccine e.g. pain or swelling at the injection site, joint pain and stiffness. Some may have more than one of these symptoms at the same time.
**26** in 100 may have moderate **(in yellow)** symptoms:<br>- **12** may have diarrhoea;<br>- **14** may get an ear infection.	**14** in 100 may have moderate **(in yellow)** symptoms:<br>- **4** may have high fever;<br>- **4** may be irritable;<br>- **1** may have swelling of salivary glands;<br>- **5** may have a non-infectious faint red rash.
**15** in 100 may have serious **(in red)** symptoms<br>- **9** may get pneumonia<br>- **5** may have measles croup<br>- **1** may have fever-induced convulsion<br>Some may be hospitalised for any of the above symptoms.	
**Rare Complications**<br>**2 in 1,000** children may have inflammation of the brain (encephalitis). Encephalitis from any reason may result in children surviving with permanent brain damage or death.	**Rare Complications**<br>Uncertain; a maximum of one child may develop encephalitis.
**3 in 1,000** children develop thrombocytopenia (tendency for bruising or bleeding).	**26 in 1,000,000** children may have thrombocytopenia.
**1 in 100, 000** children may get subacute sclerosing panencephalitis (SSPE), a late complication of measles, which causes progressive brain damage and nearly always results in death.	No children will get subacute sclerosing panencephalitis (SSPE).

Mumps

- ***Common symptoms of mumps***

These are usually mild symptoms and include fever, mild headaches, abdominal pain, loss of appetite, painful and swollen glands in the cheeks, neck or under the jaw in 7 out of 10 people. These symptoms usually go away within 10 days or so, if there is no serious complication.

- ***Complications of mumps***

These are usually serious conditions and include inflammation of the pancreas (pancreatitis), partial or complete deafness and inflammation of the brain (encephalitis), which could result in hospitalisation. Complications are more serious after puberty. Boys (after puberty) and men may experience painful, swollen testicles, which very rarely causes infertility. Mumps may cause spontaneous miscarriage during the 1st three months of pregnancy. Mumps is the commonest cause of meningitis in the UK.

Now look at the following table that compares the potential problems caused by mumps with the potential problems caused by the MMR vaccine.

Green - Common, usually mild symptoms that can be treated at home.
Yellow - Moderate complications that need medical attention but may not include hospitalisation.
Red - Serious complications that need urgent medical attention and could include hospitalisation.

**POTENTIAL RISKS IN A GROUP OF 100 CHILDREN UNDER 5 YEARS OF AGE AND ADOLESCENTS WHO GET MUMPS**	**POTENTIAL RISKS IN A GROUP OF 100 CHILDREN WHO HAVE THE MMR VACCINE**
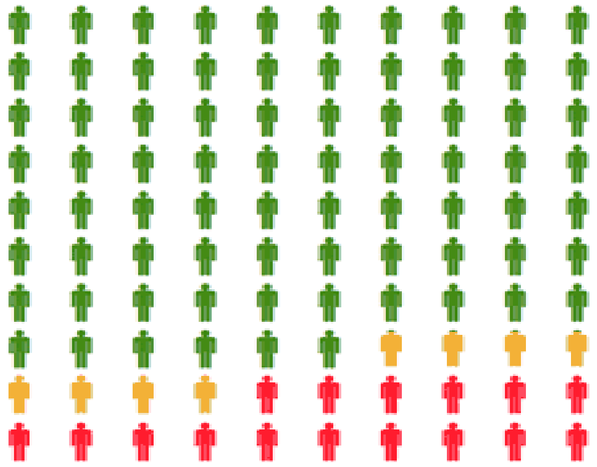<br>1/3 of children will have no symptoms. Most children will have the common and usually mild **(in green)** symptoms of mumps e.g. fever, tiredness, runny nose, loss of appetite, general aches and pains.**76** in 100 children may have swollen cheeks or swelling under the jaw.	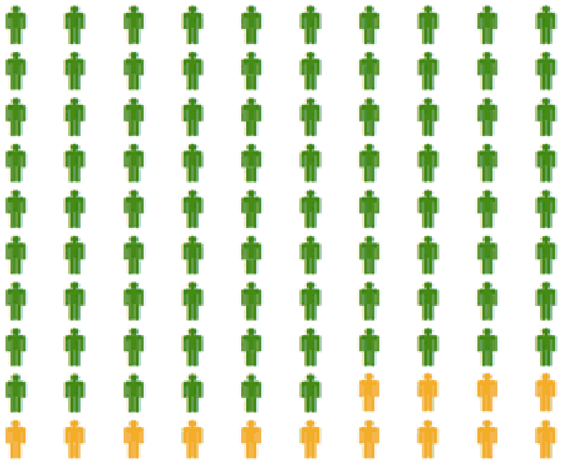<br>Most will have common and usually mild **(in green)** symptoms of the MMR vaccine e.g. pain or swelling at the injection site, joint pain and stiffness. Some may have more than one of these symptoms at the same time.
Some may have more than one of these symptoms listed above at the same time **(in yellow).**	**14** in 100 may have moderate **(in yellow)** symptoms:<br>- **4** may have high fever;<br>- **4** may be irritable;<br>- **1** may have swelling of salivary glands;<br>- **5** may have a non-infectious faint red rash.
**16** in 100 may have serious **(in red)** symptoms:<br>- **4** may have inflamed pancreas (pancreatitis) causing pain and vomiting;<br>- **8** may have mild and temporary inflammation of the lining of the brain (aseptic meningitis);<br>- **4** may have temporary hearing loss.	
**Rare Complications**<br>**3 in 1,000** children may have inflammation of the brain (encephalitis).<br>**1 in 20,000** children may have permanent deafness, usually on one side.	**Rare Complications**<br>**25 to 34 in 100,000** children may have fever-induced fits or convulsions.<br>**1 in 1 million** children may have inflammation of the brain (encephalitis).<br>Up to **4 in 1 million** children may get a severe allergic reaction (anaphylaxis).<br>**4 in 100,000** children may have a temporary tendency for bruising or bleeding (thrombocytopenia)

Rubella

Common symptoms of rubella

These are usually mild symptoms and include fever, swollen glands, joint pain and a red rash around the ears and neck.

- ***Complications of rubella***

These are usually serious conditions and include a tendency to bleed or bruise (thrombocytopenia), deafness and inflammation of the brain (encephalitis), which could result in hospitalisation. Congenital rubella (which means rubella infection of an unborn child) is a very serious condition. If a woman catches rubella during the first 3 months of her pregnancy, the virus almost always causes serious birth defects (congenital abnormalities) in her unborn child. This can include deafness, blindness, heart defects or damage to the brain.

Now look at the following table that compares the potential problems caused by rubella with the potential problems caused by the MMR vaccine.

Green - Common, usually mild symptoms that can be treated at home.
Yellow - Moderate complications that need medical attention but may not include hospitalisation.
Red - Serious complications that need urgent medical attention and could include hospitalisation.

**POTENTIAL RISKS IN A GROUP OF 100 CHILDREN UNDER 5 YEARS OF AGE WHO GET RUBELLA**	**POTENTIAL RISKS IN A GROUP OF 100 CHILDREN WHO HAVE THE MMR VACCINE**
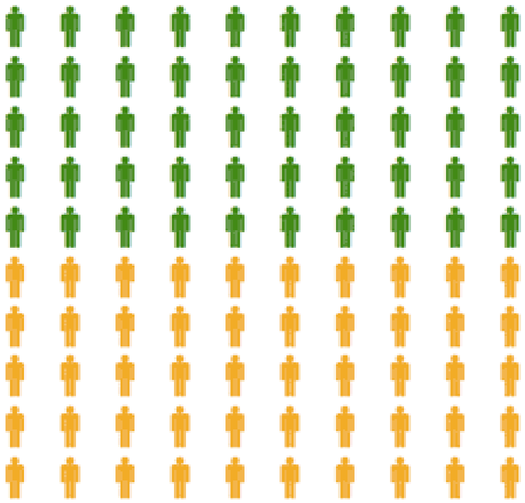<br>50 children will have the common and usually mild **(in green)**symptoms of rubella e.g. fever, tiredness, sore eyes, rash, painful and swollen glands.	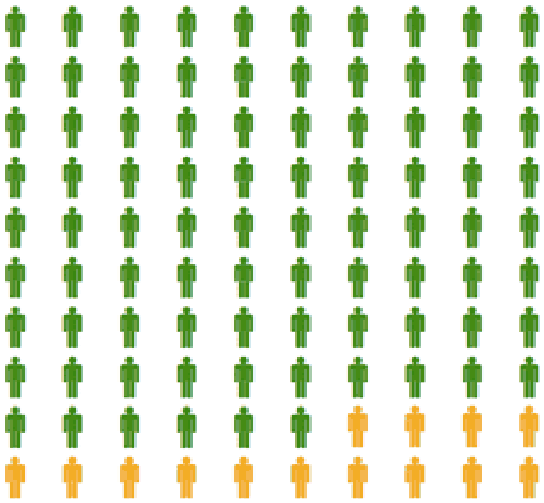<br>Most will have common and usually mild **(in green)** symptoms of the MMR vaccine e.g. pain or swelling at the injection site, joint pain and stiffness. Some may have more than one of these symptoms at the same time.
Some may have more than one of these symptoms listed above at the same time **(in yellow)**	**14** in 100 may have moderate **(in yellow)** symptoms<br>- **4** may have high fever<br>- **4** may be irritable<br>- **1** may have swelling of salivary glands<br>- **5** may have a non-infectious faint red rash
**Rare Complications**<br>**1 in 3,000** children may have a temporary tendency for bruising or bleeding (thrombocytopenia)**.**<br>**1 in 6,000** children may have inflammation of the brain (encephalitis). Encephalitis from any reason may result in children surviving with permanent brain damage or death.<br>**90% of babies** infected in the 1st trimester (i.e. 1st three months of pregnancy) will have major birth defects (congenital abnormalities) such as deafness, blindness, heart defects or damage to the brain.<br>Very rarely, a person may get degenerative brain inflammation i.e. progressive rubella pan-encephalitis.	**Rare Complications**<br>**25 to 34 in 100,000** children may have fever-induced fits or convulsions.<br>**1 in 1 million** children may have inflammation of the brain (encephalitis). Encephalitis from any reason may result in children surviving with permanent brain damage or death.<br>Up to **4 in 1 million** children may get a severe allergic reaction (anaphylaxis). Anaphylaxis from any reason, may result in death.<br>Up to **4 in 100, 000** children may have a temporary tendency for bruising or bleeding (thrombocytopenia)

Fear Correction

- IN THIS EXPERIMENT YOU WILL SEE SOME OF THE CONSEQUENCES YOU MAY FACE BY CHOOSING TO NOT VACCINATE YOUR CHILD.
- MEASLES, MUMPS, AND RUBELLA ARE SERIOUS DISEASES. A COMBINED SHOT — CALLED *MMR* VACCINE — CAN PREVENT THESE DISEASES. ASK YOUR DOCTOR FOR MORE INFORMATION.
- NOW PLEASE TURN THE PAGE.

MEASLES

- The measles virus can be spread very easily.
- Even being in the same room with a person with measles is enough to catch the disease.
- Symptoms include a rash, fever, cough and watery eyes.
- Measles also can cause pneumonia, brain damage, seizures or death.

Image available at:
http://www.idph.state.il.us/about/immunepics/measles.htm

MUMPS

- The mumps virus causes fever, headaches and swollen salivary glands under the jaw.
- Children who get mumps may develop meningitis (inflammation of the covering of the brain and spinal cord) and encephalitis (inflammation of the brain).
- Mumps can also result in permanent hearing loss.

Image available at:
http://www.idph.state.il.us/about/immunepics/mumps.htm

RUBELLA

- The rubella virus usually causes mild sickness with fever, swollen glands and a rash that last about three days.
- But, if a pregnant woman gets rubella, she can lose her baby, or the baby can be born blind, deaf, mentally retarded, with heart defects or other serious problems.

Image available at:
http://www.idph.state.il.us/about/immunepics/rubella.htm

Control

- IN THIS EXPERIMENT YOU WILL READ SOME FACT SHEETS CONTAINING TIPS TO HELP PREVENT MEDICAL ERRORS AND GET SAFER HEALTHCARE.

- YOU CAN GO THROUGH THEM AT YOUR OWN PACE.

- BEGIN ON THE NEXT PAGE.

Materials for this intervention were drawn from:

https://www.ahrq.gov/sites/default/files/wysiwyg/patients-consumers/care-planning/errors/20tips/20tips.pdf [AHRQ Pub. No. 110089]

https://archive.ahrq.gov/patients-consumers/care-planning/errors/5steps/5steps.pdf [Pub No. AHRQ 04-M005]
